# Manufacturing of Polymer–Metal Composite by Fused Filament Fabrication: Adhesion of PLA and PETG on Aluminum

**DOI:** 10.3390/polym17162210

**Published:** 2025-08-13

**Authors:** Miguel Campos-Jurado, Óscar Rodríguez-Alabanda, Guillermo Guerrero-Vacas

**Affiliations:** Department of Mechanical Engineering, Higher Polytechnic School, University of Córdoba, Rabanales University Campus, 14014 Córdoba, Spain; orodriguez@uco.es (Ó.R.-A.); guillermo.guerrero@uco.es (G.G.-V.)

**Keywords:** aluminum–magnesium alloy, metal–polymer bonding, metal–polymer composite, PETG, PLA, shear test, surface treatment, tensile test

## Abstract

The formation of metal–polymer composites by 3D printing PLA and PETG onto EN AW-5182 H111 aluminum substrates without the use of adhesives was investigated. Four surface treatments were evaluated on the metal substrate (fine sanding, coarse sanding, abrasive blasting, and acid etching), over which a polymer primer—prepared from PLA and PETG solutions—was applied. Subsequently, test specimens were fabricated using the same polymer through material extrusion (MEX) with filaments. Adhesion strength between the printed polymer and the metal substrate was assessed through perpendicular tensile, lap shear, and three-point bending tests. The 16-condition experimental matrix combined surface treatment, primer thickness, and bed temperature and was replicated for each test. Peak tensile and shear strengths confirmed the effectiveness of the proposed strategy, with PETG consistently showing a higher interfacial performance than PLA. ANOVA analysis identifies primer layer thickness (*p* = 0.023) and loading type (*p* = 0.031) as statistically significant variables. The results suggest that either abrasive or acid pretreatment, combined with a primer thickness ≥ 80 µm and moderate bed temperatures (65 °C for PLA and 90 °C for PETG), enables the fabrication of robust metal–polymer joints, which are particularly resistant to shear stress and suitable for industrial applications.

## 1. Introduction

The integration of polymeric and metallic materials into structural components has gained increasing relevance in industrial sectors such as automotive, aerospace, and advanced manufacturing [1,2,3]. Metal–polymer composites combine the mechanical strength of metals with the versatility and lightness of polymers, enabling enhanced component performance and expanding the range of functional applications [4,5]. However, bonding these dissimilar materials remains challenging due to their differing physicochemical properties, such as surface energy [6], thermal expansion coefficients, and chemical compatibility [7]. Traditional joining methods include chemical adhesion using structural adhesives and mechanical fastening with rivets or screws, both of which present significant limitations. While chemical adhesion requires complex surface treatments and the use of substances that may hinder material recyclability [8], mechanical joints generate stress concentrations that can reduce component lifespan [9].

To overcome these drawbacks, several advanced joining techniques have been developed, including friction welding [10], spot welding [11], over molding via injection molding on metal substrates [12], laser-assisted bonding [12,13,14], and cold spray deposition [15]. Nonetheless, these methods still face barriers in terms of cost, scalability, and compatibility with certain polymers. In this context, additive manufacturing (AM)—particularly material extrusion (MEX) with filaments, as defined by ASTM/ISO 52900:2021—has emerged as an innovative alternative for fabricating metal–polymer composites without conventional adhesives [16]. For consistency with widespread practice in the additive manufacturing community, the equivalent term fused filament fabrication (FFF) will be used throughout this paper while acknowledging that MEX is the standardized terminology.

Compared with other additive manufacturing (AM) techniques for thermoplastics—such as selective laser sintering (SLS), stereolithography (SLA), or binder jetting—material extrusion (MEX) provides a unique advantage for polymer–metal bonding studies. Recent reviews on multi-material AM note that SLS and SLA rely on polymer powders or photopolymers that require post-processing and limit direct polymer–metal contact, while binder jetting introduces binders that can obscure interfacial behaviour and complicate adhesion evaluation [2,17]. MEX extrudes molten filament directly onto the treated aluminium, enabling the polymer to wet and anchor into the microtextures generated by abrasive blasting or acid etching. This direct deposition under moderate processing temperatures provides an optimal scenario to evaluate how surface treatments and primer layers influence adhesion strength without interference from binders, residual powders, or resin chemistries.

Beyond PLA and PETG, other thermoplastics have also been printed onto metals and ceramics using MEX. Studies report ABS bonded to aluminium and steel [18], PA12 on metallic inserts [7], and even PC combined with aluminium alloys [19]. These examples confirm that polymer choice strongly affects adhesion mechanisms and supports MEX as a versatile route for polymer–metal hybrids.

3D printing enables the controlled deposition of polymers onto metallic substrates, offering new possibilities for tailoring and optimizing interfacial adhesion [20]. Recent studies have explored these possibilities by combining FFF printing with mechanically textured aluminum substrates. Bechtel et al. (2022) demonstrated that grit-blasted aluminum surfaces provide effective micro-mechanical interlocking for FFF-printed PLA and PETG, resulting in robust polymer–metal joints [21].

Despite recent advances, the experimental landscape remains fragmented: the studied metal–polymer pairs, surface preparation methods, and testing criteria vary widely among authors, hindering direct comparisons and the establishment of universal trends [19,22]. Addressing these limitations, the present work employs a factorial experimental design that systematically varies surface treatment, primer layer thickness, and printing temperature to isolate the effect of each variable and propose transferable guidelines for industrial application.

**Figure 1:**
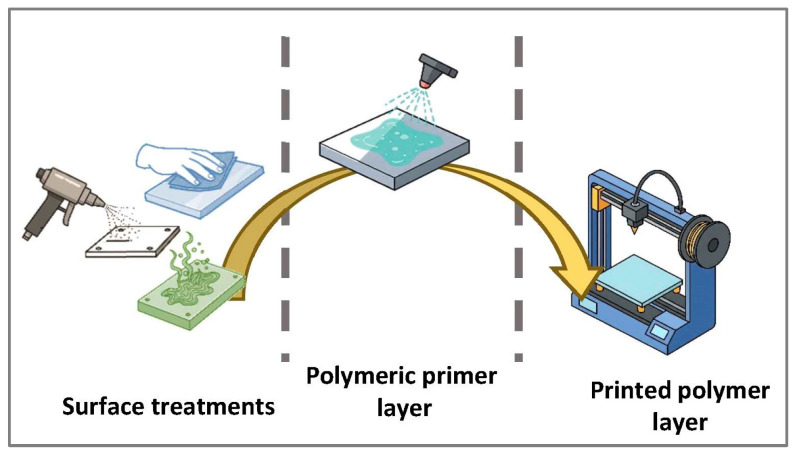
Fabrication process of metal–polymer hybrid test specimens, involving surface treatment, priming with a polymer solution layer on the aluminum substrate, followed by 3D printing of polymer materials.

The success of this strategy largely depends on the surface preparation of the metal substrate, as well as on printing parameters, which directly affect the quality and strength of the bond [7,18]. The adhesion of PLA and PETG printed onto EN AW-5182 H111 aluminum–magnesium alloy substrates using FFF 3D printing with a Creality Ender 3 V2 printer (Shenzhen Creality 3D Technology Co., Ltd., Shenzhen, China) has been investigated. To enhance adhesion, several surface treatments—sanding, abrasive blasting, and chemical etching—are evaluated. Previous research has shown that acid-based treatments and anodizing processes generate porous oxide layers on aluminum that improve adhesion by providing chemical bonding sites and additional interfacial anchoring for molten polymers [23]. Tensile and shear tests analyzed the influence of these treatments on bond strength. In addition, the effect of a polymeric primer layer applied prior to deposition was assessed, exploring its impact on primer layer uniformity and the reduction of structural defects. Figure 1 schematically illustrates the fabrication process of the test specimens used in this study.

An optimized and replicable process is proposed for fabricating metal–polymer composite materials by 3D printing, eliminating the need for chemical adhesives and enhancing adhesion through controlled surface treatments. The results define more efficient and sustainable manufacturing strategies with potential applicability in industrial settings [24]. Furthermore, the findings advance the understanding of polymer–metal interfacial interactions and support future developments in hybrid material systems via additive manufacturing.

## 2. Materials and Methods

### 2.1. Materials

The metallic substrates used in this study correspond to the aluminum–magnesium alloy EN AW-5182 H111 (Broncesval S.L., Valencia, Spain), a material widely used in industrial applications due to its high formability and mechanical strength. The typical chemical composition of EN AW-5182 H111 aluminum, which belongs to the 5000 series (Al-Mg), is provided in Table 1, in accordance with the EN 573-3 standard.

To enhance the adhesion of the primer layer, the metallic substrates were subjected to specific surface treatments prior to polymer deposition. Three surface preparation techniques were employed: mechanical sanding, chemical etching, and abrasive particle blasting. The polymeric materials used in this study were polylactic acid (PLA) and polyethylene terephthalate glycol (PETG), both in filament form for 3D printing (Smart Materials 3D Printing S.L., Jaén, Spain). PLA was selected for its biodegradability and ease of processing, while PETG was chosen for its superior mechanical strength and impact resistance.

### 2.2. Experimental Design

To analyze the influence of aluminum surface preparation, primer layer thickness, and print bed temperature on the adhesion of polymeric primer layers, a factorial experiment was designed using two materials—polylactic acid (PLA) and polyethylene terephthalate glycol (PETG)—both applied via fused filament fabrication (FFF) 3D printing. The study considered different combinations of surface treatments—fine sanding, coarse sanding, abrasive blasting, and acid etching—along with variations in the thickness of the polymer primer, which was prepared by dissolving the polymer in methylene chloride and applying one or two layers to achieve thicknesses ranging from approximately 41 to 116 µm. Additionally, two levels of bed temperature were considered: 65 °C and 85 °C for PLA and 90 °C and 110 °C for PETG. These ranges were selected to bracket the glass transition temperature (Tg) of each polymer—around 55–65 °C for PLA [26] and 75–85 °C for PETG [27]—and to evaluate how thermal input affects adhesion and interfacial behavior. Lower bed temperatures promote dimensional stability and prevent excessive flow, while higher temperatures enhance wetting of the treated aluminum and diffusion of the molten polymer into the microtextures created by blasting or etching, improving mechanical anchoring and primer layer integration.

For each experimental combination, at least four independent replicates were fabricated and evaluated through three types of destructive mechanical tests: perpendicular tensile testing to assess normal peel strength; lap shear testing to evaluate resistance to tangential stress; and three-point bending delamination testing to characterize behavior under deformation. The corresponding tables summarize the experimental matrix defined for each polymer and the tested combinations.

The experimental matrix for each polymer is summarized in Table 2.

### 2.3. Surface Treatment of Aluminum Substrates

The aluminum samples were subjected to the following surface treatments to enhance the adhesion of the polymeric primer layer:

- Mechanical sanding: P80 and P180 grit sandpapers (INDASA Abrasivos Ibérica S.A., Barcelona, Spain) were used to generate two different surface roughness levels. Sanding was performed by rubbing intensively for 10 s in two perpendicular directions on the substrate.
- Abrasive blasting: Fine brown corundum F100 (106–150 µm) supplied by Abshot (Abshot Tecnics S.L., Cervelló, Barcelona, Spain) was used with a CAT-990 particle blasting system (MetalWorks, Barcelona, Spain). The abrasive was applied at 40 mm and a pressure of 0.4 MPa for 15 s.
- Chemical etching: Samples were immersed in a 3M hydrochloric acid (HCl) solution (Alcoholes del Sur S.A., Córdoba, Spain) for 15 min.

After treatment, all samples were cleaned with ethanol and dried in a convection oven at 50 °C for 2 h.

### 2.4. Application of Primer Layer

Before the 3D printing of the printed polymer layer, a primer layer was applied to improve material adhesion. Solutions of PLA and PETG were prepared by dissolving the polymers in industrial-grade methylene chloride (99%) (Alcoholes del Sur S.A., Córdoba, Spain) at two concentrations: a 100% saturated solution and a 75% diluted solution. The solutions were applied using two methods: immersion and spray application. In the first stage, samples were immersed in the solution and subsequently dried in a convection oven at 50 °C for 2 h. Given that dichloromethane has a low boiling point of 40 °C, at atmospheric pressure and a high vapor pressure (≈47 kPa at 20 °C) [28], heating ensured complete and uniform evaporation of the thicker solvent layer deposited during immersion and eliminated any solvent trapped in surface asperities. In the second stage, the primer was applied by controlled spraying in very thin layers. In this case, the solvent evaporated rapidly at room temperature due to its high volatility, leaving a dry and uniform primer layer without the need for additional heating. The final thickness of the primer layer was measured using a portable thickness gauge, Leptoskop 2042 (Karl Deutsch Prüf- und Messgerätebau GmbH & Co. KG, Wuppertal, Germany), recording the average value of five measurements per sample.

### 2.5. Preparation of Metal–Polymer Hybrid Specimens

The primer polymer layer, completing the fabrication of the metal–polymer hybrid specimens, was applied by fused filament fabrication (FFF) using a Creality Ender 3 V2 printer (Shenzhen Creality 3D Technology Co., Ltd., Shenzhen, Guangdong, China). To ensure good adhesion, the aluminum specimens were secured to the printer’s aluminum build plate using adhesive tape applied only to their edges, so that the treated surface remained fully exposed and in direct contact with the heated bed. Printing was carried out directly onto the aluminum surface, with no intermediate glass, PEI sheet, or coating layer. The printing parameters used are listed in Table 3.

No printing issues or dimensional deviations were observed for either PLA or PETG under these parameters, confirming that the selected settings ensured consistent layer deposition, accurate geometries, and repeatability across all printed specimens.

**Figure 2:**
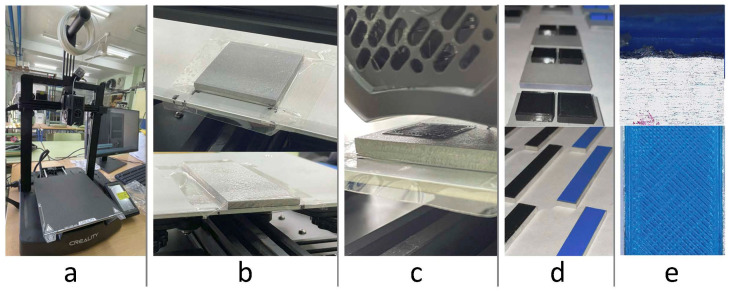
(**a**) Creality Ender 3 V2 printer; (**b**) aluminum samples on the heated bed; (**c**) direct PLA/PETG deposition; (**d**) printed specimens for tensile, shear, and bending tests; (**e**) close-ups of surface finish and polymer–metal adhesion.

Figure 2 presents the experimental setup, including the Creality Ender 3 V2 printer (Shenzhen Creality 3D Technology Co., Ltd., Shenzhen, China), aluminum samples fixed to the heated bed, the direct deposition of PLA/PETG onto the treated substrates, the printed geometries for tensile, shear, and bending tests, and close-ups of surface finish and polymer–metal adhesion. These images provide a clear overview of the configuration used during printing and visually document the direct deposition process onto the aluminum surfaces.

The aluminum plates were indirectly preheated by conduction from the printer’s heated build plate, which was set at the selected printing temperatures (90–110 °C for PETG and 65–85 °C for PLA). As the aluminum remained in direct contact with the heated bed, it reached thermal equilibrium with the set temperature prior to polymer deposition. In preliminary trials, a thermographic camera (FLIR ONE^®^ Pro for iOS, FLIR Systems Inc., Santa Barbara, CA, USA) was used to verify that the aluminum surface achieved the target temperature before the printing process commenced. No instances of warping or detachment were observed during any of the printing trials.

Each metallic substrate was designed with specific geometry and dimensions according to the type of mechanical test to be conducted (tensile, shear, and three-point bending). For both tensile and shear tests, identical aluminum substrates were used, measuring 50 mm × 50 mm × 5 mm, while for the three-point bending test, substrates with dimensions of 16 mm × 100 mm × 5 mm were employed. The deposition layer designs were created using SolidWorks 2023 (Dassault Systèmes S.A., Boston, MA, USA), generating three distinct geometrical models tailored to the selected mechanical tests. These designs were subsequently processed using slicing software UltiMaker Cura 5.10.1 (UltiMaker B.V., Geldermalsen, The Netherlands) to produce the appropriate G-code for the printer.

**Figure 3:**
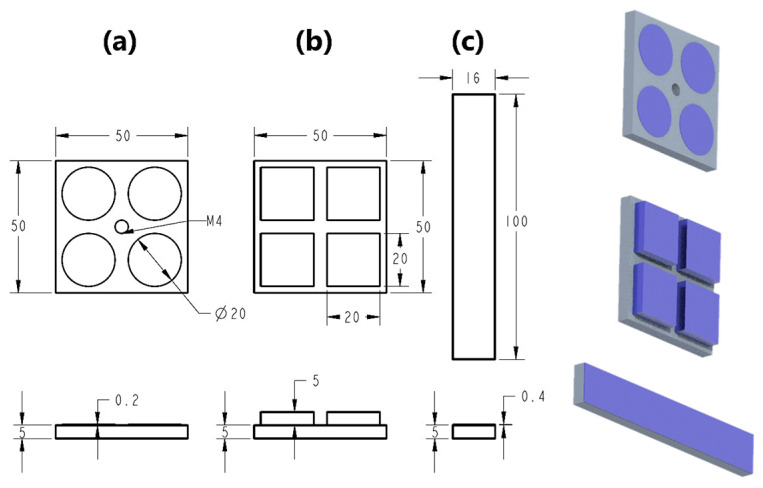
Morphology and dimensions of the test specimens used for tensile (**a**), shear (**b**), and three-point bending (**c**) tests.

For the tensile (pull-off) test, a circular disk measuring 20 mm in diameter and 0.2 mm in thickness was designed and replicated four times on each substrate. In the shear (lap shear) test, a square prism of 20 mm × 20 mm × 5 mm was used and deposited four times per substrate. Finally, for the three-point bending test, a rectangular prism of 16 mm × 100 mm × 0.4 mm was designed and deposited once, matching the dimensions of the corresponding substrate. The morphology and dimensions of the test specimens are shown in Figure 3.

### 2.6. Mechanical Testing

**Figure 4:**
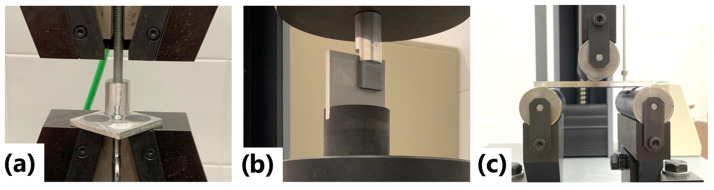
Experimental designs: (**a**) tensile test, (**b**) shear test, and (**c**) three-point bending test.

The experimental campaign was carried out in two phases to evaluate the strength of the metal–polymer bond under different loading conditions. In Phase 1, destructive mechanical tests were performed, including tensile and shear tests, to analyze the adhesion of the primer polymer layer to the metallic substrate under various combinations of surface treatment and priming conditions. Based on the results obtained in this first stage, Phase 2 focused on the most favorable configurations, which were selected for evaluation through three-point bending delamination tests, to assess the resistance of the printed polymer layer under tensile stresses induced by bending. Figure 4 illustrates the experimental designs for the three mechanical tests—tensile, shear, and three-point bending—showing the fixtures and specimen configurations used in each case.

#### 2.6.1. Tensile Test (Pull-Off)

The tensile test was carried out using a Servosis ME 405-5 testing machine (Servosis S.L., Madrid, Spain), applying a force perpendicular to the specimen surface until failure of the joint. A cylindrical metallic fixture with an M8 thread was bonded to the printed polymer layer, using Loctite Super Glue-3 Power Gel instant adhesive (Henkel Ibérica S.A., Barcelona, Spain), to ensure uniform load transfer during the tensile test.

#### 2.6.2. Shear Test (Lap Shear)

The shear test was also carried out on the Servosis ME 405-5 testing machine (Servosis S.L., Madrid, Spain), applying a tangential force to evaluate the resistance to shear stress. A custom-designed fixture was employed to ensure a homogeneous distribution of the applied load.

#### 2.6.3. Three-Point Bending Test

Test specimens were designed with dimensions of 100 mm × 16 mm × 5.4 mm, with the polymer layer located on the lower face, subjected to tensile stress during deformation. The test was performed on the Servosis ME 405-5 machine, configured for simple bending with a support span of 80 mm. Displacement was applied incrementally from 0 mm (0°) to 17 mm (54°), 27 mm (89°), and 37 mm (120°), monitoring the integrity of the printed polymer layer at each stage. The onset of delamination was assessed using scanning electron microscopy (SEM) and visual inspection. After reaching the final deformation level (37 mm), the central region of the specimen (the tensioned area during bending) was brushed to verify the post-test adhesion of the printed polymer layer.

### 2.7. Morphological Characterization and Statistical Analysis

#### 2.7.1. Surface Characterization

A scanning electron microscope JEOL JSM 7800F Prime (JEOL Ltd., Tokyo, Japan) was used to analyze the surface morphology before and after the printed polymer layer. Additionally, surface roughness was measured using a contact profilometer, Mitutoyo SJ-201 (Mitutoyo Corporation, Kawasaki, Japan).

#### 2.7.2. Statistical Analysis Methodology

Statistical analysis was performed using Minitab software, version 19 (Minitab LLC, State College, PA, USA), applying ANOVA and contour plots to evaluate the influence of surface treatments on the mechanical strength.

## 3. Results

### 3.1. Surface Characterization of Pretreated Aluminum with Polymer Primer Solution

**Figure 5:**
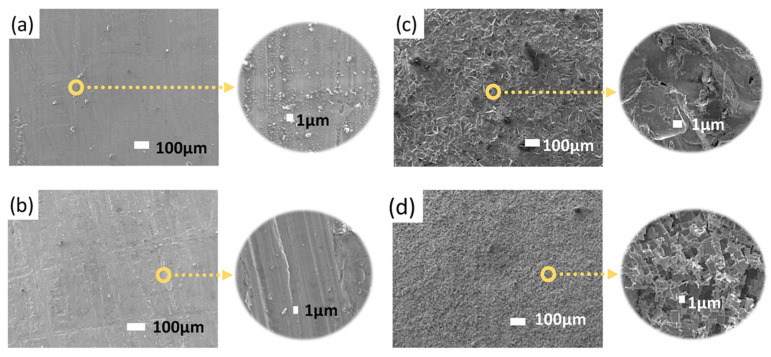
Morphology of the aluminum surface after (**a**) no pretreatment, (**b**) sanding, (**c**) abrasive treatment, and (**d**) chemical etching.

Figure 5 shows the surface morphology of the aluminum substrate before and after the application of different surface treatments, as observed by scanning electron microscopy (SEM). Figure 5a shows the untreated aluminum surface, where surface impurities and intermetallic particles are heterogeneously distributed. These surface defects may negatively affect the adhesion of the printed polymer, highlighting the need for appropriate pretreatment. Figure 5b presents the morphology after mechanical sanding, showing a controlled roughness pattern dependent on the abrasive used. The roughness generated in this process contributes to improved adhesion by increasing the effective contact area. In Figure 5c, corresponding to the surface treated by abrasive particle blasting, a greater topographical heterogeneity is observed, with randomly distributed microcavities. Finally, Figure 5d shows the surface after chemical etching with HCl, where selective etching has produced a more homogeneous porous texture compared to mechanical treatments.

Table 4 shows the roughness of the EN AW-5182 H111 aluminum substrates after different mechanical and chemical treatments. Characterization was performed using a Mitutoyo SJ-201 contact profilometer (Mitutoyo Corporation, Kawasaki, Japan), measuring the arithmetic mean roughness (Ra), root mean square roughness (Rq), and ten-point mean roughness (Rz).

The as-received condition was measured only for reference purposes and was not included in subsequent mechanical tests. Fine sanding generated a relatively low and controlled roughness, with average values of Ra ≈ 0.69 µm, Rz ≈ 5.27 µm, and Rq ≈ 0.89 µm. In contrast, coarse sanding significantly increased the surface roughness, reaching Ra ≈ 3.17 µm, Rz ≈ 21.93 µm, and Rq ≈ 4.09 µm. Abrasive blasting resulted in the highest roughness values among the tested treatments, with Ra ≈ 4.25 µm, Rz ≈ 30.22 µm, and Rq ≈ 5.37 µm, producing a heterogeneous surface with randomly distributed microcavities. Acid etching, on the other hand, yielded comparable roughness levels (Ra ≈ 3.70 µm, Rz ≈ 23.71 µm, Rq ≈ 4.60 µm) but produced a more homogeneous porous texture compared to the mechanically treated surfaces. These variations in surface roughness reflect the distinct topographies generated by each preparation method, which were subsequently analyzed in relation to the adhesion performance of the printed polymer layer.

**Figure 6:**
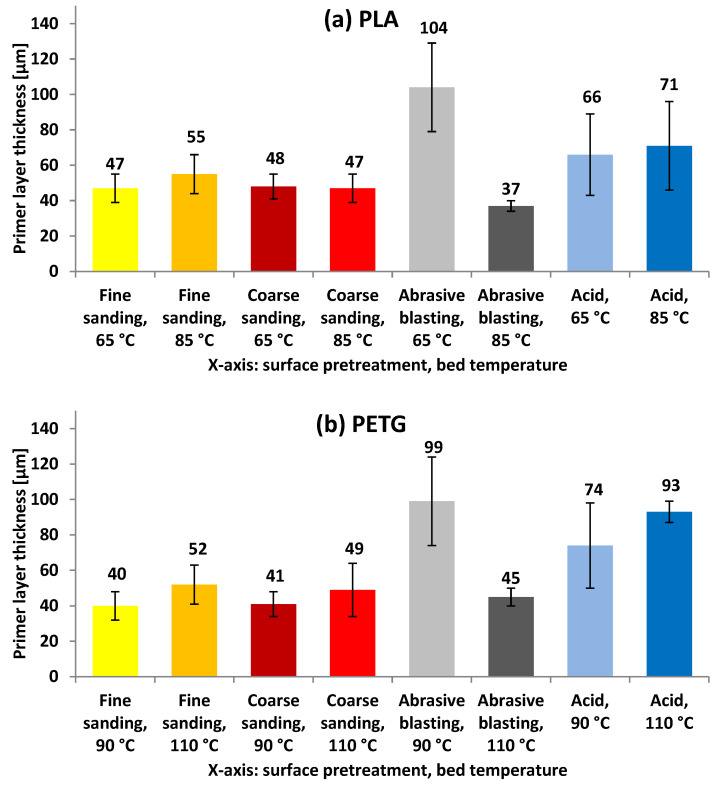
Average thicknesses of the polymer primer layer applied to the test specimens for (**a**) PLA and (**b**) PETG as a function of the surface treatment of the aluminum substrate and the bed temperature used during 3D printing.

These surface modifications play a key role in the interaction between the printed polymer layer and the metallic substrate, directly influencing the quality of the bond. Prior to the deposition of the molten polymer, the specimens were primed with a polymer solution applied to the treated surfaces. Figure 6 shows the average thickness of this transitional primer layer between the pretreated metal and the deposited polymer for the different experiments conducted in both Phase 1 and Phase 2 of the study.

The characterization of primer layer thicknesses, obtained after applying polymer solutions on EN-AW 5182 H111 aluminum substrates, was carried out with the aim of including this parameter as an experimental variable in subsequent analyses. The measured values ranged between 37 and 104 µm for PLA and between 40 and 99 µm for PETG, providing a sufficient basis for exploring their potential influence on metal–polymer adhesion. The bed temperature used during printing did not affect these values, as measurements were taken prior to the deposition of the molten polymer. This experimental approach allows the subsequent analysis of two distinct scenarios: on the one hand, the possible existence of an optimal primer layer thickness range for each surface treatment and, on the other, the tolerance or sensitivity of each treatment to variations in primer layer thickness.

### 3.2. Evaluation of Metal–Polymer Bond Strength in Phase 1

**Figure 7:**
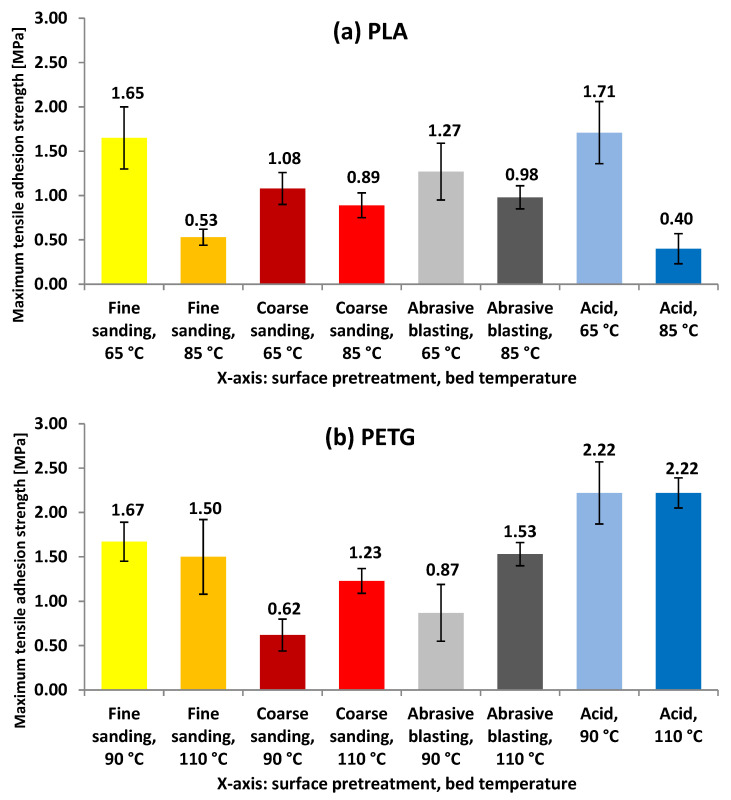
Maximum tensile force before adhesive failure in test specimens for (**a**) PLA and (**b**) PETG as a function of the aluminum substrate surface treatment and the bed temperature used during 3D printing.

In Phase 1, pull-off and lap shear tests were performed to evaluate the bond strength between the printed polymer layer and the metallic substrate under different combinations of surface treatment and primer application. Figure 7 presents the average tensile strength values obtained in the pull-off tests, comparing the adhesion performance of PLA and PETG polymers on pretreated aluminum surfaces.

In the case of PLA, a noticeable dependence on both surface treatment and bed temperature was observed. The highest tensile adhesion strength was obtained with acid etching at 65 °C (1.71 MPa), indicating strong interfacial compatibility under normal loading conditions. Coarse sanding and abrasive blasting at 65 °C also yielded moderate values (1.08 MPa and 1.27 MPa, respectively), whereas increasing the bed temperature to 85 °C led to a significant decrease in performance across all surface treatments. The lowest value was recorded for acid etching at 85 °C (0.40 MPa), suggesting a potential loss of interfacial compatibility or thermal degradation of the primer–polymer interface. For PETG, the results were more consistent across temperature variations. The maximum shear strength was achieved with acid etching at 90 °C and 110 °C (5.94 MPa and 4.45 MPa, respectively), indicating a strong compatibility between the etched surface and the polymer. Abrasive blasting and fine sanding also delivered intermediate values, with fine sanding at 90 °C reaching 1.24 MPa and abrasive blasting at 110 °C reaching 1.58 MPa. Coarse sanding, in contrast, was the least effective method for PETG, with values below 1.60 MPa under all conditions.

**Figure 8:**
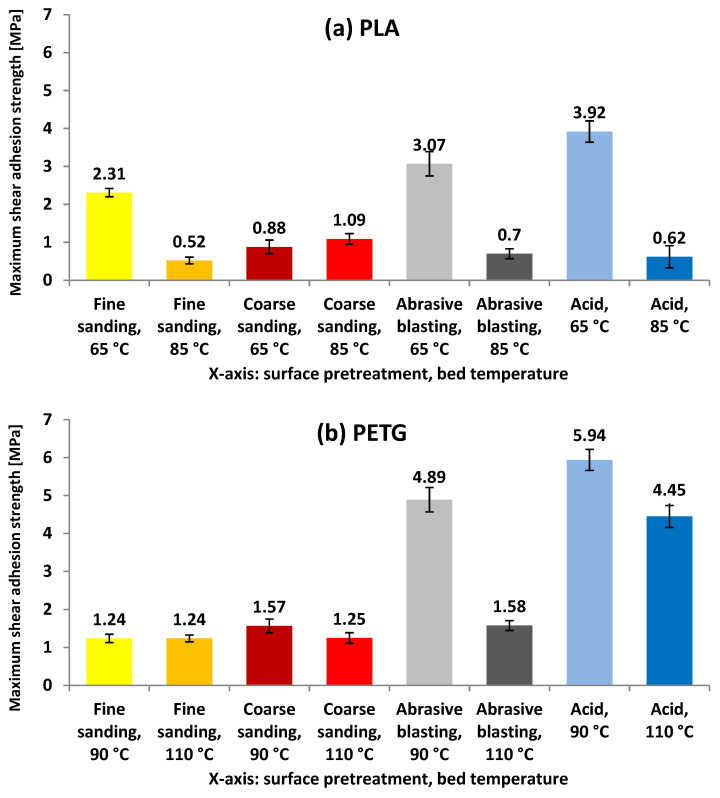
Maximum shear force before adhesive failure applied to the test specimens for (**a**) PLA and (**b**) PETG as a function of the surface treatment of the aluminum substrate and the bed temperature used during 3D printing.

Figure 8 shows the maximum shear strength prior to adhesion failure measured for PLA and PETG under different surface treatment and bed temperature combinations.

In the shear tests, the results show a clear trend favoring the more aggressive surface treatments (abrasive blasting and acid etching) combined with moderate bed temperatures. For PLA, the highest shear adhesion strength was obtained with acid etching at 65 °C (3.92 MPa), followed by abrasive blasting at the same temperature (3.07 MPa), confirming that elevated surface roughness together with a restrained printing temperature promotes resistance to tangential loading. By contrast, combinations with higher bed temperatures (85 °C) or less intensive treatments, such as fine sanding, yielded much lower values, dropping to a minimum of 0.52 MPa (fine sanding, 85 °C). For PETG, the highest strengths also occurred in acid-etched samples, with 5.94 MPa at 90 °C and 4.45 MPa at 110 °C. Abrasive blasting at 90 °C followed closely (4.89 MPa), standing out as the most effective non-chemical option. These figures indicate higher shear resistance for PETG than for PLA and suggest the better interfacial compatibility of PETG with chemically activated or topographically rough surfaces. In both materials, acid treatment at low-to-moderate bed temperature emerges as the most efficient combination, whereas fine sanding (particularly at the higher temperature) provides the lowest performance, pointing to possible thermal mismatch effects or reduced mechanical interlocking.

### 3.3. Evaluation of Delamination Under Bending in Phase 2

**Figure 9:**
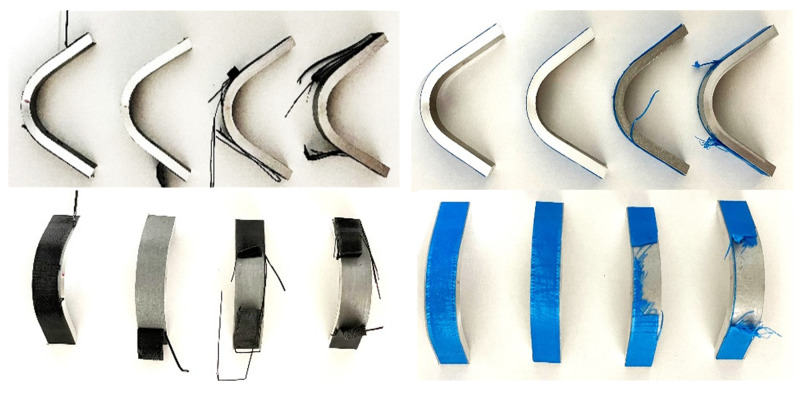
Representative examples of bending test specimens at their final deformation state.

After analyzing the results of the tensile and shear tests conducted in Phase 1, the most promising combinations of surface treatment and primer were selected for validation under complex loading conditions. Specifically, the delamination resistance was evaluated by means of three-point bending tests, in which the polymer layer was positioned on the underside of the sample and subjected to tensile stresses induced by bending. Representative examples of specimens after the bending tests are shown in Figure 9.

The success criterion was defined as the ability of the printed polymer layer to remain adhered to the metallic substrate after completing the full deformation cycle and the final mechanical brushing. All tested specimens were classified as “pass,” “fail,” or “quasi-pass.” Based on the number of iterations (four per experiment), a success rate was calculated for each test configuration. A specimen was considered “pass” if no polymer fiber detachment was observed in the central section of the sample. It was considered “quasi-pass” when detachment affected less than 25% of the central section (2.5 mm). Any other case was classified as “fail.” In the analysis, each “quasi-pass” specimen was given half the weight of a “pass” specimen.

**Figure 10:**
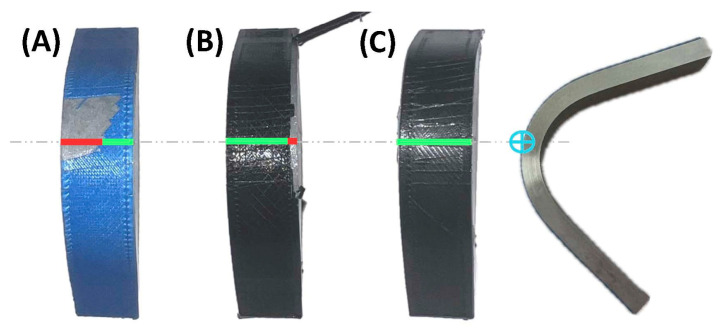
Examples used for interpreting the tested specimens: (**A**) fail, (**B**) quasi-pass, (**C**) pass. Green lines indicate the region of the maximum stress plane where the polymer remained adhered to the metallic substrate after testing, whereas red lines indicate the region of the maximum stress plane where the polymer detached from the substrate.

Figure 10 shows three representative examples of specimens to illustrate the interpretation criteria used: specimen “A” was classified as fail, “B” as quasi-pass, and “C” as pass.

Table 5 summarizes the percentage of specimens that maintained the integrity of the bond at the end of the test.

These results confirm that delamination is strongly influenced by the specific combination of surface treatment, bed temperature, and polymer–substrate interaction. This behavior contrasts with the strong performance of PETG under acid etching in tensile and shear tests, suggesting that bending resistance is more affected by the overall stiffness and induced stresses than by interfacial compatibility alone. The poor bending/peel performance of acid-etched PETG–Al joints, in contrast with their high lap-shear strength, arises from switching the load from shear (Mode II) to opening (Mode I); under peel, interfacial toughness drops sharply, and the thin porous-oxide layer produced by HCl etching fails brittlely [6,8]

Conversely, in the PLA samples, both surface treatments (abrasive and acid etching) at 65 °C exhibited comparable performance, with a 50% success rate. This indicates a higher sensitivity of PLA to printing temperature and the type of mechanical deformation applied, which should be considered in applications involving bending loads.

Table 6 summarizes the best-performing combinations of polymer, surface treatment, and bed temperature for each mechanical test. This table consolidates the tensile, shear, and bending results presented in Section 3.2 and Section 3.3, highlighting the conditions that yielded the highest adhesion performance and the key observations for each scenario.

### 3.4. Statistical Analysis

To identify the factors that significantly influence the mechanical strength of the tested materials, a General Linear Model (GLM) was applied using Minitab software. The maximum stress supported by each specimen (Stress [MPa]) was considered as the response variable while including, as categorical factors, the material type (PETG or PLA), the type of mechanical load (tensile or shear), and the surface treatment (fine sanding, coarse sanding, abrasive blasting, or acid etching). Continuous covariates also included bed temperature, surface roughness parameters Ra, Rz, and Rq, and primer layer thickness.

The input data are included in the statistical analysis shown in Table 7.

During model fitting, the surface treatment factor was automatically removed due to collinearity with the roughness variables, suggesting statistical redundancy. Table 8 below presents the analysis of variance (ANOVA) for the fitted model.

The analysis shows that the primer layer thickness [µm] and the type of mechanical loading are the only factors with a statistically significant influence on the response variable (mechanical strength), with *p*-values below 0.05. In contrast, neither the type of material, nor the test temperature, nor the surface roughness parameters (Ra, Rz, or Rq) exhibited statistically significant effects. This suggests that, at least within the range of tested conditions, the variation in strength does not substantially depend on the temperature or the base material but rather on the way the load is applied and the functional thickness of the primer layer. Table 9 presents the coefficients of the fitted model.

The Variance Inflation Factor (VIF) values associated with the roughness parameters were extremely high, especially Ra and Rq, which indicate a strong collinearity between these variables, as expected. The final model equation can be expressed in simplified form as follows:

Stress [MPa] = 1.34 − 0.0094∙Temperature [°C] + 11.7∙Ra [µm] + 0.117∙Rz [µm] − 9.9∙Rq [µm] + 0.01954∙primer layer thickness [µm] − 0.002∙Material_PETG + 0.002∙Material_PLA + 0.446∙Loading_Shear stress − 0.446∙Loading_Tensile stress(1)

The adjusted coefficient of determination of the model was 33.04%, indicating a moderate ability to explain the observed experimental variability. We acknowledge that the roughness parameters Ra, Rq, and Rz show strong multicollinearity, as reflected by their high VIF values. The current model was kept unchanged to preserve the integrity of the original analysis, but this issue will be addressed in future work by considering variable reduction strategies or composite indices.

## 4. Conclusions

The results confirm that FFF printing on EN AW-5182 H111 aluminum substrates pretreated by abrasive blasting or acid etching generates microtextures up to Ra ≈ 4.25 µm and Rz ≈ 30.2 µm, which retain primer layers ranging from 37 to 104 µm in PLA and 40–99 µm in PETG, consistent with previous findings showing that grit-blasted aluminum provides effective mechanical interlocking for FFF-printed polymers [21]. Under these conditions, the tensile strength reached a maximum of 1.71 MPa (PLA, acid etching, with a bed temperature of 65 °C) and 2.22 MPa (PETG, acid etching, with bed temperatures of 90 and 110 °C), while in shear tests, peak values of 5.94 MPa (PETG, acid etching, 90 °C) and 3.92 MPa (PLA, acid etching, 65 °C) were recorded. In three-point bending tests, bond integrity was preserved in 62.5% of PETG specimens with aluminum substrates treated by abrasive blasting and a bed temperature of 90 °C, in 50% of PLA specimens treated with either abrasive or acid pretreatment at 65 °C, and in only 12.5% of PETG specimens pretreated with acid at 90 °C. This low percentage is notable, given the excellent performance of this combination in tensile and shear tests. The difference highlights the sensitivity of joints to the nature of applied stress and suggests that bending resistance may be limited by polymer stiffness and the printed polymer layer’s ability to deform without delaminating.

The ANOVA results show that the primer layer thickness (*p* = 0.023) and the loading mode (*p* = 0.031) are the only factors with a statistically significant influence on the mechanical performance, whereas surface roughness and bed temperature did not show significant effects. Nevertheless, the mechanical results reveal a clear trend: moderate bed temperatures (65 °C for PLA and 90 °C for PETG) provided the highest adhesion, while higher settings (85 °C for PLA and 110 °C for PETG) consistently reduced tensile and shear strength. This decrease can be explained by excessive melt flow at high heat, which produces thinner and less structured polymer layers with weaker mechanical interlocking [29], local relaxation and partial softening of PLA near its glass transition [26], and the build-up of residual stresses in PETG, which promotes microcracks and delamination upon cooling [19]. Therefore, it can be concluded that a pretreatment involving either abrasive blasting or acid etching, combined with a primer layer thickness of ≥80 µm and moderate bed temperatures (PLA at 65 °C and PETG at 90 °C), enables the formation of robust metal–polymer joints, with particularly high performance under shear loading, supporting its industrial applicability.

Although acid etching provided the highest tensile and shear strengths for both polymers, its performance under bending diverged significantly. In the case of PETG, the bending success rate was only 12.5%, which can be attributed to the brittle aluminum oxide film formed during acid treatment, a behavior similar to the oxide fragility observed in anodized aluminum surfaces reported by [23]. This thin layer tends to microcrack under flexural stress, compromising interfacial integrity and triggering early delamination. PLA, in contrast, showed a higher tolerance to bending under the same pretreatment (50% success), likely due to its lower stiffness and better compliance with the oxide layer, which allowed minor substrate deformation without causing premature failure.

The methodology shows strong potential for industrial transfer, particularly in sectors seeking lightweight hybrid solutions that avoid adhesives. The aluminum–PLA/PETG system is already valuable for rapid prototyping of jigs, housings, decorative panels, and orthotic supports and could evolve toward end-use parts where moderate loads and temperatures are expected. Incorporating higher performance polymers, such as ABS, in future work could broaden the range of viable products, potentially extending this approach to more demanding markets like automotive interiors and heavy-duty consumer goods.

Overall, this study lays the groundwork for the development of functional and adaptable metal–polymer hybrid joints aligned with the demands of advanced manufacturing.

## Figures and Tables

**Table 1:** Chemical composition of EN AW-5182 H111 [25].

Element	Symbol	% by Weight (Approx.)
Aluminum	Al	Remainder (approx. 94.8–95.5%)
Magnesium	Mg	4.0–5.0%
Manganese	Mn	0.2–0.5%
Iron	Fe	≤0.35%
Silicon	Si	≤0.20%
Chromium	Cr	≤0.10%
Copper	Cu	≤0.15%
Zinc	Zn	≤0.25%
Titanium	Ti	≤0.10%
Others (each)	-	≤0.05%
Others (total)	-	≤0.15%

**Table 2:** Experimental matrix for PLA and PETG.

Nº	Surface Treatment	Bed Temperature [°C] PETG	Bed Temperature [°C] PLA
1	fine sanding	90	65
2	fine sanding	110	85
3	coarse sanding	90	65
4	coarse sanding	110	85
5	abrasive	90	65
6	abrasive	110	85
7	acid	90	65
8	acid	110	85

**Table 3:** Characteristic parameters used in 3D printing.

Printing Parameters	Units	PETG	PLA
Nozzle temperature	°C	235	210
Bed temperature	°C	90/110	65/85
Printing speed	mm/s	40	40
Fan speed	%	Variable	Variable
Layer height	mm	0.2	0.2
Wall thickness	mm	1.6	1.6
Infill density	%	100	100
Infill pattern	-	Lineal	Lineal
Nozzle diameter	mm	0.4	0.4
Build plate material	-	Aluminum	Aluminum
Chamber type	-	Open	Open

**Table 4:** Surface roughness values of the aluminum specimens according to the applied surface pretreatments.

Surface Treatment	Arithmetic Mean Roughness (Ra) [µm]	Root Mean Square Roughness (Rz) [µm]	Ten-Point Mean Roughness (Rq) [µm]
As received	0.17 ± 0.07	1.06 ± 0.43	0.21 ± 0.09
fine sanding	0.69 ± 0.08	5.27 ± 0.90	0.89 ± 0.09
coarse sanding	3.17 ± 0.49	21.93 ± 3.50	4.09 ± 0.64
abrasive	4.25 ± 0.21	30.22 ± 2.14	5.37 ± 0.30
acid	3.70 ± 0.25	23.71 ± 1.27	4.60 ± 0.21

**Table 5:** Percentage of success in the tested specimens.

Surface Pretreatment, Bed Temperature [°C]	Polymer	Successful [%]
Abrasive, 65	PLA	50
Acid, 65	PLA	50
Abrasive, 90	PETG	62.5
Acid, 90	PETG	12.5

**Table 6:** Summary of the best-performing conditions for PLA and PETG across mechanical tests.

Polymer	Surface	Bed [°C]	Tensile [MPa]	Lap Shear [MPa]	Bending [% Success]	Key Observations
PLA	Fine sanding	65 °C	1.65	2.31	-	Moderate adhesion from low roughness; tested only in tensile and shear
PLA	Abrasive blasting	65 °C	1.27	3.07	50%	Rough surface improved shear adhesion and gave 50% bending success
PLA	Acid etching	65 °C	1.71	3.92	50%	Chemical anchoring boosted tensile and shear, but brittle oxide layer limited bending
PETG	Fine sanding	90 °C	1.67	1.24	-	Low roughness gave weak shear adhesion; bending not assessed
PETG	Abrasive blasting	90 °C	0.87	4.89	62.50%	Best PETG condition; strong interlocking led to top shear and bending results
PETG	Acid etching	90 °C	2.22	5.94	12.5%	Strong bonding produced top tensile/shear, but brittle oxide film reduced bending success

**Table 7:** Data for statistical analysis.

Loading	Material	Surface	Bed Temp. [°C]	Ra [µm]	Rz [µm]	Rq [µm]	Primer Layer Thickness [µm]	Stress [MPa]
Tensile	PETG	fine sanding	90	0.21	1.58	0.273	35.36	1.67
Tensile	PETG	fine sanding	110	0.21	1.58	0.273	57.66	1.5
Tensile	PETG	coarse sanding	110	1.22	7.99	1.57	35.32	1.23
Tensile	PETG	coarse sanding	90	1.22	7.99	1.57	43.5	0.62
Tensile	PETG	abrasive	90	4.35	31.14	5.517	111.3	0.87
Tensile	PETG	abrasive	110	4.35	31.14	5.517	41.16	1.53
Tensile	PETG	acid	110	3.76	24.21	4.675	87.4	1.86
Tensile	PETG	acid	90	3.76	24.21	4.675	52.18	1.65
Tensile	PLA	fine sanding	65	0.21	1.58	0.273	55.5	1.65
Tensile	PLA	fine sanding	85	0.21	1.58	0.273	48.02	0.53
Tensile	PLA	coarse sanding	85	1.22	7.99	1.57	41.84	0.89
Tensile	PLA	coarse sanding	65	1.22	7.99	1.57	55.98	1.08
Tensile	PLA	abrasive	65	4.35	31.14	5.517	120.54	1.27
Tensile	PLA	abrasive	85	4.35	31.14	5.517	36.86	0.98
Tensile	PLA	acid	85	3.76	24.21	4.675	107.98	0.4
Tensile	PLA	acid	65	3.76	24.21	4.675	31.12	1.71
Shear	PETG	fine sanding	90	0.21	1.58	0.273	37.86	2.31
Shear	PETG	fine sanding	110	0.21	1.58	0.273	61.76	0.52
Shear	PETG	coarse sanding	110	1.22	7.99	1.57	40.6	0.88
Shear	PETG	coarse sanding	90	1.22	7.99	1.57	51.56	1.09
Shear	PETG	abrasive	90	4.35	31.14	5.517	76.14	3.07
Shear	PETG	abrasive	110	4.35	31.14	5.517	37.5	0.7
Shear	PETG	acid	110	3.76	24.21	4.675	97.72	3.92
Shear	PETG	acid	90	3.76	24.21	4.675	33.2	0.62
Shear	PLA	fine sanding	65	0.21	1.58	0.273	45.42	1.24
Shear	PLA	fine sanding	85	0.21	1.58	0.273	46.12	1.24
Shear	PLA	coarse sanding	85	1.22	7.99	1.57	37.64	1.57
Shear	PLA	coarse sanding	65	1.22	7.99	1.57	61.7	1.25
Shear	PLA	abrasive	65	4.35	31.14	5.517	121.44	4.89
Shear	PLA	abrasive	85	4.35	31.14	5.517	47.9	1.58
Shear	PLA	acid	85	3.76	24.21	4.675	117.59	5.94
Shear	PLA	acid	65	3.76	24.21	4.675	98.48	4.45

**Table 8:** Analysis of variance of the GLM model.

Source	DF	Adj SS	Adj MS.	*p*-Value
Temperature [°C]	1	0.2786	0.2786	0.634
Ra [µm]	1	1.2866	1.2866	0.310
Rz [µm]	1	0.1028	0.1028	0.772
Rq [µm]	1	0.9938	0.9938	0.371
Primer layer thickness [µm]	1	7.0499	7.0499	0.023
Material	1	0.0001	0.0001	0.994
Loading	1	6.3302	6.3302	0.031
Error	24	28.7429	1.1976	
Total	31	55.4487		

**Table 9:** Estimated coefficients of the model.

Term	Coefficient	SE Coef.	T-Value	*p*-Value	VIF
Constant	1.34	1.84	0.73	0.474	
Temperature [°C]	−0.0094	0.0195	−0.48	0.634	2.60
Ra [µm]	11.7	11.3	1.04	0.310	10,125.89
Rz [µm]	0.117	0.400	0.29	0.772	608.64
Rq [µm]	−9.9	10.9	−0.91	0.371	14,776.51
Primer layer thickness [µm]	0.0195	0.0081	2.43	0.023	1.44
Material (PETG vs. PLA)	±0.002	0.310	±0.01	0.994	2.57
Loading (Shear/Tensile)	±0.446	0.194	±2.30	0.031	1.00

## Data Availability

The original contributions presented in this study are included in the article. Further inquiries can be directed to the corresponding author.
